# Cholera

**Published:** 1873-07

**Authors:** 


### CHOLERA.

This disease has broken out in many parts of our country, and without the usual warning. Heretofore, when it has prevailed, notice was given of its approach. We have heard of it in all its previous visits as spreading out of India into Russia, then pursuing its march across Europe until it reached France and England, and thence from some of their ports being brought to our shores. This is the history that has been given of all the outbreaks of the disease on our continent up to this time; but now it seems to have broken out in one of our own cities before invading Western Europe. About two months ago cases of what bore a strong resemblance to malignant cholera, in symptom and termination, were reported in New Orleans. A

steamer leaving that city for Cincinnati lost a number of deck-hands or passengers on its way. Not long after it was announced in New Orleans, cases of the disease made their appearance at Memphis, and at points on the river below. There was a difference in opinion among physicians as to the character of the malady, but all agreed that it terminated fatally in a few hours when suffered to run on unchecked, and that its subjects died in collapse, having had rice-water discharges and cramps.

The latest accounts from Memphis show that the disease is abating there, but after a month the mortality still exceeded ten a day. Nashville has suffered severely in all former visitations of cholera, and the pestilence in the last few days has shown a malignity in that city not exhibited up to this time at any other point in the country. On June 20th, seventy-three deaths were reported there from cholera, and the mortality ranged from forty to seventy during the week from the 15th to the 22d, The disease has also appeared at Gallatin Hartsville, Lebanon, and Murfreesboro, in Tennessee, and at Paducah, Indianapolis, and Cincinnati; but in none of these places has it assumed an epidemic form. After Nashville, Gallatin, on the railroad twenty-six miles from that city, has been most seriously afflicted.

There has been a singular unwillingness among physicians to admit that the disease was cholera wherever it has appeared. The cases were first referred to cholera morbus, and set down to some indiscretion in diet. A few cases have occurred in Louisville, but a general skepticism prevails in the profession as to there having been cases of genuine cholera. We believe that it is true Asiatic or malignant cholera which is now spreading over the country, and which has been so fatal in a few places. We have no doubt that three or four sporadic cases have originated in Louisville; but a peculiarity of the disease exhibited at most points is, that it shows no disposition to become epidemic. No new cases have followed those which more than a fortnight ago terminated in death, after a few hours' illness, in this city.

In Nashville some very noteworthy facts have been brought out by the present epidemic. The disease has been limited to a few localities, and a very large proportion of the victims have been negroes. On one day forty-nine out of seventy-three were colored, and on another forty-eight out of fifty-nine. When the mortality exceeded seventy a day the city proper was comparatively healthy; hardly any citizens had the disease. The infected localities are creek bottoms, subject to overflow, where the thriftless inhabitants live in crowded, badly-ventilated huts, and drink water from feeble springs or wells excavated in the limestone rock. In Memphis and Gallatin, too, the mortality among the colored population has been quite out of proportion to that of the whites.

Physicians, wherever the disease has yet appeared, concur in the statement that it is entirely manageable, if cases receive timely treatment. Negroes rarely apply for medical aid before cramps have supervened, when collapse is impending, and the condition of things nearly hopeless. At the penitentiary in Nashville ninety of the convicts were reported ill at one time, most of them with cholera, but all in a promising condition.

When cholera is prevailing, every case of diarrhœa should be treated as the premonitory symptom of the disease. An opiate will generally check it, but if the case is not of the mildest character, we should not be content with simply arresting the evacuations; we should not rest satisfied until we had brought about discharges of another kind by calomel. The indications of cure appear to us to be to check the watery discharges, keep up the vital powers of the patient, and secure dark, consistent evacuations.

The Atlantic cable brings accounts of cholera in some of the cities of Germany, and there is reason to apprehend that it will become epidemic in Europe during the warm season. Having originated on our continent, we may infer that it has at last become naturalized, and in future may look for it to break out from time to time, as it has done in New Orleans after an incubation of seven years. Its progress during the hot months immediately before us will be watched with intense interest.—*The American Practitioner.*

---

Removal of Plaster of Paris Bandage.—A strong solution of common salt causes the plaster to crumble, so that the bandage can easily be cut. It also quickly cleanses the hands of the operator from the gritty article used for securing immobility.

